# MSN8C: A Promising Candidate for Antitumor Applications as a Novel Catalytic Inhibitor of Topoisomerase II

**DOI:** 10.3390/molecules28145598

**Published:** 2023-07-24

**Authors:** Jie-Bin Ou, Wei-Hao Huang, Xing-Zi Liu, Guo-Yao Dai, Lu Wang, Zhi-Shu Huang, Shi-Liang Huang

**Affiliations:** 1 School of Pharmaceutical Sciences, Sun Yat-sen University, Guangzhou 510006, China; huangwh28@mail2.sysu.edu.cn (W.-H.H.); liuxz7@mail2.sysu.edu.cn (X.-Z.L.); daigy8@mail2.sysu.edu.cn (G.-Y.D.); wanglu79@mail2.sysu.edu.cn (L.W.); ceshzs@mail.sysu.edu.cn (Z.-S.H.); 2 Department of Pharmacy, Sun Yat-sen Memorial Hospital, Sun Yat-sen University, Guangzhou 510120, China; oujieb3@mail.sysu.edu.cn; 3 Guangdong Provincial Key Laboratory of New Drug Design and Evaluation, Guangzhou 510120, China

**Keywords:** topoisomerase II, antiproliferation, catalytic inhibitor, MSN8C, mansonone E

## Abstract

MSN8C, an analog of mansonone E, has been identified as a novel catalytic inhibitor of human DNA topoisomerase II that induces tumor regression and differs from VP-16(etoposide). Treatment with MSN8C showed significant antiproliferative activity against eleven human tumor cell lines in vitro. It was particularly effective against the HL-60/MX2 cell line, which is resistant to Topo II poisons. The resistance factor (RF) of MSN8C for Topo II in HL-60/MX2 versus HL-60 was 1.7, much lower than that of traditional Topo II poisons. Furthermore, in light of its potent antitumor efficacy and low toxicity, as demonstrated in the A549 tumor xenograft model, MSN8C has been identified as a promising candidate for antitumor applications.

## 1. Introduction

Topoisomerase II (Topo II) is an essential enzyme for the survival of eukaryotes [1]. It can mediate transient DNA double-strand breakage and rejoining and plays an important role in DNA replication, transcription, recombination and chromosome segregation. Traditional Topo II inhibitors, such as etoposide (VP-16), mainly function by forming a Topo II–DNA–drug ternary complex or intercalating into DNA, affecting the progression of the replication fork, leading to failed DNA replication and cytotoxicity, which have demonstrated certain clinical efficacy [2]. These drugs are referred to as Topo II poisons. Despite their long-term clinical use, the application of these drugs is limited due to their ability to induce DNA breaks, activate DNA repair pathways and cause drug resistance [3]; they may also exhibit genotoxicity resulting from DNA mismatches [4,5]. A new class of Topo II catalytic inhibitors mainly function by inhibiting the hydrolysis of ATP by Topo II ATPase, restricting enzyme conformation to the DNA reconnection stage but preventing its release from the clamp structure and affecting the enzyme’s unwinding of DNA and re-entering the cycle. This process does not induce DNA cleavage, and thus does not trigger the DNA repair system. In principle, these inhibitors have lower toxicity and fewer side effects, and have become a research hotspot in recent years in the field of Topo II inhibitors [6,7,8].

**Figure 1:**
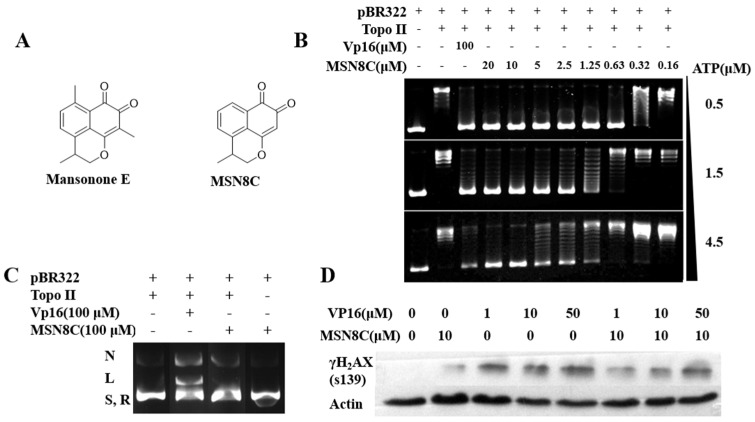
(**A**) The structures of mansonone E and MSN8C. (**B**) Results from experiments investigating the activity of MSN8C on Topo II-mediated DNA relaxation at different ATP concentrations. Row 1 is the pBR322 control; Row 2 is Topo II and pBR322; Row 3 is Topo II, pBR322 and 100 μM VP16; Row 4–11 is Topo II and pBR322 at the indicated MSN8C concentrations and at different ATP concentrations. (**C**) Effect of MSN8C on Topo II–DNA cleavage complex. Row 1 is Topo II and pBR322; Rows 2–3 are the cleavage activity assay of Topo II for compound VP16 and MSN8C at 100 μM, respectively. Row 4 is only pBR322 and MSN8C; N—nicked DNA, L—link DNA, R—relaxed DNA and S—supercoiled DNA are shown in the figure. (**D**) The expression of S139 phosphorylated histone H_2_AX (γH_2_AX) analyzed via Western blot.

Mansonone E (3,6,9-trimethyl-2,3-dihydrobenzo[de]chromene-7,8-dioneis), a type of sesquiterpene O-naphthoquinone compound, is rare in natural products. It is mainly found in the trunks of plants belonging to the *Ulmaceae* family, such as the *Ulmus carpinifolia* and *Ulmus laevis*, commonly known as the winged elm and European white elm, respectively. It is a type of plant toxin and is secreted by plants as a defense mechanism against bacteria or fungal infections [9]. The content of this substance is very low in normal plant tissues. Literature reports have shown that mansonone E has various pharmacological activities, such as antimicrobial [9], anti-MRSA [10] and antitumor activities [11,12]. However, the natural antitumor activity of mansonone E is still not ideal, and total synthesis is challenging with a low overall yield [13], making it difficult to apply. In previous studies [13,14], we conducted a series of structural modifications on mansonone E/F and obtained a preferred compound, MSN8C (Figure 1A), with excellent comprehensive properties. Further studies on MSN8C confirmed that it is a new type of Topo II catalytic inhibitor with good antitumor activity in vitro and in vivo. These research findings provide a foundation for the clinical application of MSN8C and offer insights into the development of more effective Topo II inhibitors. Therefore, MSN8C, as a preferred compound, has the potential to be developed for the treatment of tumors.

## 2. Results and Discussion

### 2.1. MSN8C as a Topo II Catalytic Inhibitor

In this study, Topo II-mediated DNA relaxation experiments were conducted. As shown in Figure 1B, MSN8C at concentrations ranging from 0.16 μM to 20 μM completely inhibited the activity of Topo II at ATP concentrations of 0.5, 1.5 and 4.5 mM. The inhibitory concentration of MSN8C positively correlated with the ATP concentration. Generally, the effect of Topo II poisons is not dependent on the ATP concentration [15]. This result suggests that MSN8C may inhibit the activity of Topo II by competing for the binding site of ATP and may be a Topo II catalytic inhibitor.

Topo II poisons and catalytic inhibitors can be distinguished using Topo II-mediated negative supercoil pBR322 DNA cleavage assays. During its catalytic cycle, Topo II covalently binds to DNA, forming a Topo II–DNA covalent complex. Common Topo II poisons, such as VP-16, can trap and stabilize this complex, forming a ternary complex that behaves as linear DNA (link DNA) in agarose gel electrophoresis, resulting in DNA breakage. However, linear DNA bands similar to VP-16 were not observed even though 100 μM of MSN8C was used (Figure 1C). Nicking DNA and linear DNA did not appear in the system without the enzyme, suggesting that MSN8C did not affect the topology of DNA. These results indicate that MSN8C is not a Topo II poison but a Topo II catalytic inhibitor. In addition, γ-H_2_AX is a major marker protein related to DNA damage. We investigated whether MSN8C has an effect on DNA double-strand breaks by examining γ-H_2_AX expression levels. As shown in Figure 1D, increasing concentrations of the positive drug VP-16, which acts as a Topo II poison, led to an increase in the expression of γ-H_2_AX, indicating that VP-16 induces DNA double-strand breaks. In contrast, the expression of γ-H_2_AX was significantly attenuated in the presence of MSN8C compared to VP-16, suggesting that MSN8C does not cause DNA double-strand breaks at the cellular level (Figure 1D). Moreover, MSN8C can also significantly weaken the production of γ-H_2_AX by VP-16. Combined with the results of pBR322 DNA cleavage assays, it indicates that MSN8C can prevent supercoiled DNA from entering the catalytic cycle to avoid capture by Topo II poisons and reduce the production of broken DNA. These results indicate that MSN8C, unlike the classical Topo II inhibitor etoposide, is not a Topo II poison but a Topo II catalytic inhibitor.

### 2.2. MSN8C Induced Apoptosis in HL-60 Cancer Cells

**Figure 2:**
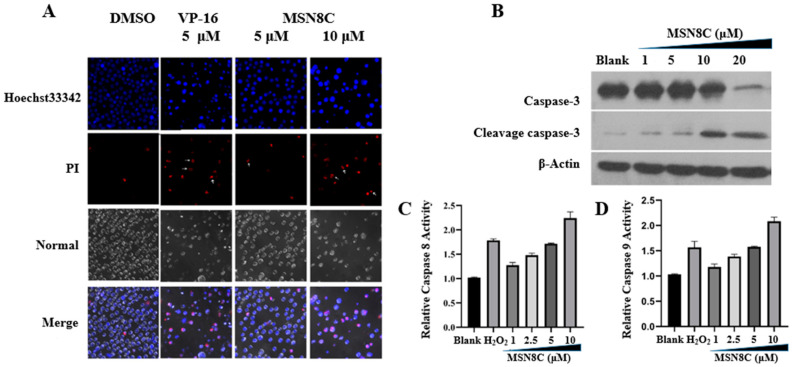
(**A**) Hoechst 33342 and PI double staining in HL-60 cells for 12 h. The magnification is ×400; (**B**) expression of caspase-3 in HL-60 treated with different concentrations of MSN8C for 24 h as detected with Western blot; (**C**) changes in caspase-8 activity in HL-60 cells after treatment with different concentrations of MSN8C for 24 h; (**D**) changes in caspase-9 activity in HL-60 cells after treatment with different concentrations of MSN8C for 24 h.

MSN8C-treated HL-60 cells were stained with Hoechst 33342 and propidium iodide (PI) prior to analysis. Figure 2A shows that cells in the control group had lower levels of Hoechst 33342 and very little PI staining, indicating intact cell morphology. When treated with VP-16 as a positive control at a concentration of 5 μM, cells absorbed more Hoechst dye and propidium iodide (PI) penetrated the nucleus, indicating incomplete cell morphology. Treatment with 5 μM MSN8C resulted in cells that had a brighter blue color than the control group, and at a concentration of 10 μM, cells showed similar characteristics to the positive control VP-16. We also identified that the expression of lysis caspases (-3, -8, -9), indicators of apoptosis, was significantly upregulated (Figure 2B) and their activity increased (Figure 2C,D) according to Western blot analysis and the caspase (-8, -9) activity assay kit after treatment with different concentrations of MSN8C for 24 h. These data suggest that MSN8C can induce cell apoptosis.

### 2.3. Effect of MSN8C on Proliferation and Growth of Tumor Cells In Vitro and In Vivo

**Figure 3:**
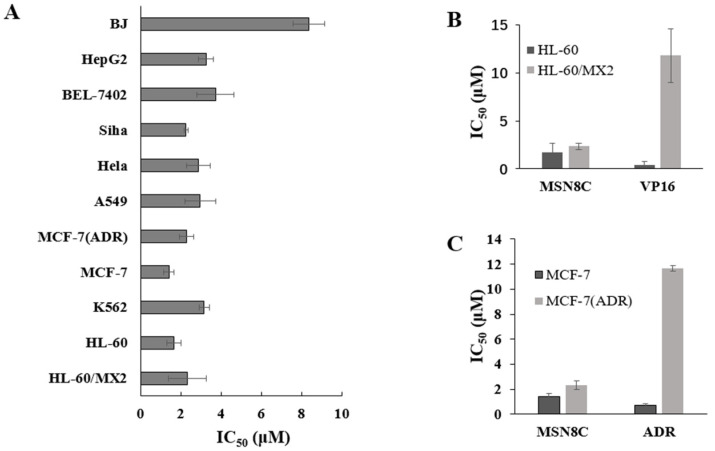
(**A**) IC_50_ values of MSN8C against cancer cells. Cancer cells were treated with the indicated concentration of MSN8C for 48 h. Cell proliferation was determined using the MTT assay; (**B**,**C**) a comparison of IC_50_ between MSN8C and VP-16/ADR in parental and drug-resistant cell lines.

The antitumor activity of MSN8C against human tumor cells was examined in vitro and in vivo. MSN8C showed potent proliferation inhibition against 11 tumor cell lines from various origins, with an average IC_50_ value of 2.60 μM and a range between 1.41 and 3.74 μM (Figure 3A and Table 1). The results show that MSN8C had a weaker antiproliferative capacity against normal cell lines (BJ) than other tumor cell lines. However, MSN8C still had relatively strong antiproliferative effects on the human breast cancer adriamycin-resistant cell line MCF-7/Adr and mitoxantrone-resistant cell line HL-60/MX2, which are normally insensitive to Topo II poisons. As shown in Figure 3B,C, the resistance factors (RF) of MSN8C against these two resistant cell lines (MCF-7/Adr vs. MCF-7 and HL-60/MX2 vs. HL-60) were 1.0 and 1.7, respectively, being significantly lower than those of the control anticancer drugs ADR (RF = 16) and VP-16 (RF = 23). These results indicate that MSN8C may be a promising new Topo II catalytic inhibitor.

**Figure 4:**
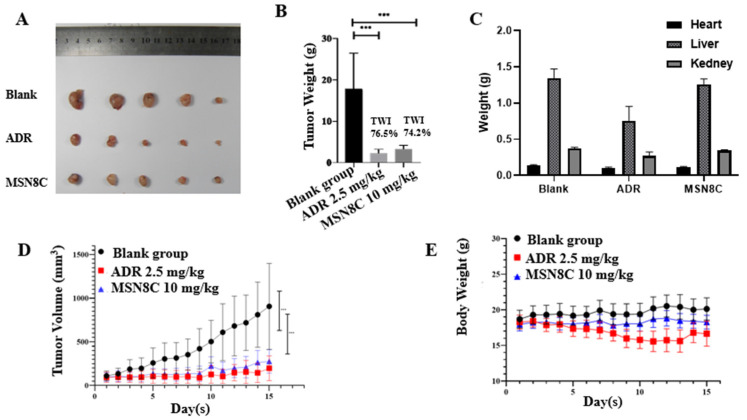
(**A**) Growth inhibition of MSN8C (10 mg/kg) and ADR (2.5 mg/kg) in human A549 nude mouse xenograft tumor model. (**B**) The tumor weight histograms of MSN8C and ADR. The TWI was calculated on the final day and compared with the blank group. All values are expressed as mean ± SD; *** *p* < 0.001 (Student’s *t*-test) indicates statistic difference. (**C**) Heart, liver and kidney weight of A549 nude mouse after 15 days of treatment with MSN8C and ADR. (**D**) Tumor volume of A549 nude mouse over 15 day of treatment with MSN8C and ADR. (**E**) Body weight of A549 nude mouse over 15 days of treatment with MSN8C and ADR.

Based on its antiproliferative effect in vitro, the compound MSN8C was further evaluated for its efficacy in vivo. MSN8C was tested in a human A549 nude mouse xenograft tumor model at a dose of 10 mg/kg. As shown in Figure 4A,D, MSN8C had similar effects compared to the control ADR (2.5 mg/kg), with both demonstrating significant inhibition of tumor growth. The tumor weight inhibition (TWI) values for ADR and MSN8C were 76.5% and 74.2%, respectively, indicating comparable levels of efficacy (Figure 4B). Significantly, the mice were found to tolerate the compound well, with minimal weight loss in the body, heart, liver and kidney, suggesting that MSN8C is less toxic than ADR (Figure 4C,E).

## 3. Materials and Methods

### 3.1. Materials

Most of the cell lines, including HeLa, Siha, Bel-7402, MCF-7, MCF-7/Adr, HepG2, HCT116 (P53^+^), A549, BJ, K562 and ECV304, were purchased from the China Center for Type Culture Collection (CCTCC, Wuhan, China), while HL-60 and HL-60/MX2 were obtained from the American Type Culture Collection (ATCC, Rockville, MD, USA). Except for the K562 and HL60 cell lines, which use RMPI-1640 as the medium, all other cell lines were cultured in Dulbecco’s modified Eagle’s medium (DMEM) containing 10% fetal bovine serum (FBS). Antibodies to γH_2_AX, Phospho-γH_2_AX (Ser139), caspase-3, cleaved caspase-3, β-actin (CST, Danvers, MA, USA) and enhanced chemiluminescence (ECL) (Pierce, Carlsbad, CA, USA) were used in this study. Topo II alpha (#HT205, Inspiralis, Norwich, UK) and pBR322 DNA (Takara, 3050, Koufushi, Japan) were purchased for inhibitory and cleavage assays of Topo II. MSN8C was synthesized according to the methods published in reference [14]. A stock solution concentration of 10 mM was prepared and stored at −20 °C before use and brought back up to room temperature at the time of use.

### 3.2. MTT Assay

The cell proliferation assay was evaluated using the MTT assay as described by Mosmann with modifications [16]. Logarithmic growth phase cells were seeded in 96-well plates at a density of about 5 × 10^3^ cells per well and allowed to adhere for 24 h before adding MSN8C. Medium containing the corresponding concentration of MSN8C was added, and blank wells (without MTT) and control wells (without drug) were set up. The plates were then incubated in a 37 °C incubator for 48 h. IC_50_ values were calculated as cell viability as measured using the MTT assay. Each test was repeated three times with a 3 × 3 setup.

### 3.3. Topo II Inhibitory Activity 

The effect of drugs on Topo II DNA relaxation was determined by measuring the conversion of negative superhelix pBR322 DNA to the dissociated state in the presence of ATP, with etoposide (VP-16) as a positive control. The relaxation assay was performed according to the manufacturer’s instructions with minor modifications. The above compounds and enzymes were mixed according to the provided protocol and incubated at 37 °C for 30 min. Then, 4 μL of a dye solution containing 1% sodium dodecyl sulfate (SDS), 0.02% bromophenol blue and 50% glycerol was added to terminate the test. The mixture was mixed with loading buffer, added to a 1% gel and electrophoresed in tris acetate EDTA (TAE) buffer (40 mM Tris-acetate, 2 mM EDTA) for 1 h. The gels were stained in 3× GelRed (Sigma-Aldrich, Darmstadt, Germany) in 0.1 M NaCl solution for 30 min. DNA bands were observed using a UV lamp, and images were captured using a gel imager.

### 3.4. Topo II DNA Cleavage Reaction Assays

The cleavage reaction assay was performed according to the manufacturer’s instructions with minor modifications. A 20 µL reaction system was prepared containing 2 µL of 0.1 μg/μL pBR322 DNA solution, 2 µL of 5 U/μL Topo II solution, 2 µL of the compound solution and 4 µL of 5× complete buffer and diluted with deionized water. The reaction mixture was incubated for 6 min at 37 °C. After incubation, 2 µL of 1% SDS was added, followed by 2 µL of 250 mM EDTA-2Na (pH 8.0) to capture the lysis intermediates. Proteinase K (0.5 µL of a 20 mg/mL solution) was added, and the reaction was incubated at 45 °C for 30 min to digest Topo II. The resulting mixture was mixed with the 10× loading buffer, added to a 1% gel and electrophoresed in TAE buffer (40 mM Tris-acetate, 2 mM EDTA) for 1.5 h. The gels were stained in 3× GelRed (Sigma-Aldrich) in 0.1 M NaCl solution for 30 min. DNA bands were observed using a UV lamp, and images were captured using a gel imager.

### 3.5. Phospho-γH_2_AX Assay and Caspase-3 and Cleaved Caspase-3 via Western Blot

HL-60 cells were grown in 6-well plates at 1 × 10^6^ cells until reaching 80% confluence. The plates were then incubated with different concentrations of MSN8C for 1 h. Cells were collected and washed twice with PBS before being lysed by adding 100 μL of solution A (50 mM Tris HCl, 300 mM NaCl, 1% Triton X-100, 10% glycerol, 1.5 mM MgCl_2_, 1 mM CaCl_2_, 1 mM phenylmethanesulfonylfluoride (PMSF) and 1% protease inhibitor cocktail). The protein concentration of each sample was determined using the BCA protein assay kit. Seventy micrograms of protein from each sample was separated with 8% or 10% SDS-PAGE and then transferred to a polyvinylidene fluoride (PVDF) membrane (Millipore, Billerica, MA, USA). The membrane was blocked with 20 mL of blocking solution (5% skim milk powder prepared by TBST), shaken for 1 h at room temperature and probed for 2–3 h with primary antibody at a dilution ratio of 1:1000. Blots were washed and shaken with horseradish peroxidase (HRP)-conjugated anti-rabbit IgG at a dilution of 1:2000 for 1 h. The protein bands were detected using ECL and visualized by a chemiluminescence substrate, with images acquired using a Tanon-4200SF gel imaging system (Shanghai, China).

### 3.6. Apoptosis Analysis

HL-60 cells (2.0 × 10^5^ cells per mL) were co-cultured with MSN8C for 12 h. After that, the cells were stained with 10 μL of Hoechst 33342 for 10 min, and then they were collected and washed twice with cold-phosphate-buffered solution (PBS). Then, the cells were resuspended in 1× binding buffer and dyed with 5 μL of propidium iodide (PI) (KeyGEN BioTECH, Nanjing, China) for 10 min in the dark. Laser confocal microscopy (LSM710, Zeiss, Oberkochen, Germany) with an excitation wavelength of 405 nm for Hoechst 33342 and 488 nm for PI was used to observe and photograph the samples. The experiments were performed with three repetitions.

### 3.7. Determination of Activity of Caspase-8 and Caspase-9 

HL-60 cells were treated with different concentrations (0, 1, 2.5, 5 and 10 μM) of MSN8C for 24 h. Cells were collected, washed twice with PBS and lysed in RIPA lysis buffer (50 mM Tris, pH 7.4, 150 mM NaCl, 1% Triton X-100, 1% sodium deoxycholate, 0.1% SDS) with 1 mM PMSF. The protein concentration was determined using a bicinchoninic acid (BCA) protein assay kit, and the lysate was supplemented with lysis buffer to achieve a final volume of 25 µL. Next, 25 µL of 2× reaction buffer (containing 0.25 µL dithiothreitol (DTT) was added to each sample, followed by 2.5 µL of caspase-8 or caspase-9 substrate, depending on the assay being performed. The samples were incubated for 4 h at 37 °C and protected from light. After incubation, the samples were analyzed using a microplate reader at a wavelength of 405 nm. The experiments were performed in triplicate.

### 3.8. Tumor Xenograft Growth Inhibition Assay

All animal experiments were conducted in accordance with the animal ethics requirements. Female BALB/c nude mice (5 weeks old) were obtained from the Laboratory Animal Center of Sun Yat-sen University and were kept under pathogen-free conditions. A549 cells were collected during their logarithmic growth and resuspended in RPMI-1640 medium at a concentration of 6.67 × 10^7^ cells/mL. Each mouse was injected subcutaneously with 1 × 10^7^ cells in the right abdomen. When the tumor volume reached approximately 100 mm^3^, the mice were randomly divided into three groups of five mice each, including a control group, an MSN8C group, and an ADR group. Mice in the treatment groups were intraperitoneally injected with the drug (MSN8C (10 mg/kg), Adriamycin (ADR) (2.5 mg/kg)) at two-day intervals for 2 weeks, while the control group was treated with an equal volume of normal saline. Tumor size and weight were measured daily, and growth curves were plotted using the mean tumor volume of all experimental groups at set time points. At the end of the treatment, animals were euthanized; their tumors, heart, liver and kidney were resected; and their weight was measured.

## 4. Conclusions

This study aimed to investigate the antiproliferative effects and potential mechanism of action of the compound MSN8C. The results demonstrate that even at high drug concentrations, MSN8C inhibited Topo II activity without causing broken DNA or inducing DNA damage markers γH_2_AX (S139) at cellular levels. These findings suggest that MSN8C is a unique Topo II catalytic inhibitor. In the enzyme inhibition experiments on ATP competition, it was implicated that MSN8C may act on the ATP site of Topo II ATPase. However, more experiments are necessary to confirm this conclusion. In in vitro and in vivo experiments, MSN8C exhibited excellent antiproliferative activity against a panel of tumor cell lines. MSN8C also exhibited some selectivity for normal cells while remaining sensitive to drug-resistant cells, such as A549/ADR and HL60/MX2. Although it was not as active as doxorubicin in animal experiments, it had a better safety profile. These findings suggest that MSN8C has the potential to become a novel Topo II catalytic inhibitor for cancer treatment. Further research is needed to explore the molecular mechanism of this compound and its pharmacological performance.

## Figures and Tables

**Table 1:** IC_50_ values of MSN8C against cancer cells. (MTT, 48 h).

Tumor Cell Lines	IC_50_ (μM)		
	MSN8C	VP16	ADR
HL-60/MX2	2.34 ± 0.95	0.44 ± 0.31	
HL-60	1.68 ± 0.35	11.82 ± 2.82	
K562	3.16 ± 0.25	12.3 ± 1.35	
MCF-7	1.41 ± 0.25		0.71 ± 0.15
MCF-7(ADR)	2.3 ± 0.35		11.65 ± 0.21
A549	2.97 ± 0.76	25.3 ± 3.23	
Hela	2.89 ± 0.59	36.2 ± 5.12	
Siha	2.27 ± 0.11	20.6 ± 1.05	
BEL-7402	3.74 ± 0.92	28.37 ± 1.21	
HepG2	3.26 ± 0.37	10.6 ± 0.53	
BJ	8.37 ± 0.77	21.23 ± 1.36	

## Data Availability

Data are contained within the article.

## Sample Availability

The compound samples used and/or analyzed during the current study are available from the corresponding author on reasonable request.
